# Limb-Specific Features and Asymmetry of Nerve Conduction Velocity and Nerve Trunk Size in Human

**DOI:** 10.3389/fphys.2020.609006

**Published:** 2020-12-03

**Authors:** Ayaka Nobue, Yoko Kunimasa, Hiromu Tsuneishi, Kanae Sano, Hiroyuki Oda, Masaki Ishikawa

**Affiliations:** ^1^Faculty of Health Sciences, Morinomiya University of Medical Sciences, Osaka, Japan; ^2^Graduate School of Sport and Exercise Sciences, Osaka University of Health and Sport Sciences, Osaka, Japan

**Keywords:** nerve conduction velocity (NCV), ultrasonography, peripheral nerve, electrical stimulation, rapid movement, reaction, nerve cross-sectional area

## Abstract

This study aimed to simultaneously examine the differences of human nerve conduction velocity (NCV) and nerve cross-sectional area (nCSA) between the upper and lower limbs and between different regions of the upper and lower limbs. Thirty healthy subjects volunteered for the study. NCV and nCSA of the ulnar and tibial nerves were measured with the dominant and non-dominant arms and the supporting and reacting legs using supramaximal electric stimulation and peripheral nerve ultrasonography at three regions for ulnar and tibial nerves, respectively. Supramaximal electric stimulation was superficially applied to the ulnar and tibial nerves at each point. These action potentials were recorded from the digiti minimi and soleus muscles for the ulnar and tibial nerves, respectively. Our results clearly showed that the NCV, nCSA, and circumference of the ulnar and tibial nerves were higher and greater in the lower limbs than in the upper limbs. The greater the circumference, the greater the nCSA for both the upper and lower limbs. However, unlike the upper limbs, the supporting leg did not have higher NCV than the reacting leg despite its greater circumference. Therefore, nCSA can be related to the circumference but not necessarily function for NCV developments of the lower limbs. These various aspects between the upper and lower limbs suggest that NCV does not depend on the nCSA sizes or upper and lower limb circumference; the results indicate the existence of limb-specific NCV but not nCSA developments.

## Introduction

The elucidation of the neuromuscular function of the human peripheral nervous system that enables rapid and accurate limb movements can be revealed by evaluating the morphology and functional characteristics of the peripheral nerves. Previous animal studies reported that trained mice had greater nerve axon diameter than the non-trained mice (Edds, 1950; Samorajski and Rolsten, 1975) and the peripheral nerve conduction velocity (NCV) was greater with the greater peripheral nerve axon diameter (Gasser and Grundfest, 1939; Hursh, 1939). In human, however, the analysis of the peripheral nerve size *in vivo* is difficult and many reports are only available on human NCV. Kim et al. (2009a) and Pawlak and Kaczmarek (2010) reported that the NCV in the trained athletes was higher in the dominant than in the non-dominant upper limbs. Hatta et al. (1995) also reported that the NCV and dominant forearm circumference were faster and greater in badminton and kendo players than those in the healthy control subjects. Consequently, they imply that the developments of the human arm circumference and its muscle size would develop the human NCV. Therefore, high resolution imaging techniques of the peripheral nerve *in vivo* are expected to prove the above speculation.

The diameter size of the peripheral nerve fiber is very small (proximately 10–30 μmm). So far, the resolution of current *in vivo* human imaging technology cannot identify the cross-sectional area (CSA) of the nerve fibers. Therefore, the factors enhancing human NCV are not fully understood in the nerve fiber level. However, recent high-resolution imaging techniques of the peripheral nerve ultrasonography allow direct measurements of the cross-sectional area of the nerve trunk (nCSA), which is a bundle of various nerve fibers. Although there is no evidence for a correlation between nCSA and either axon diameter or NCV, this peripheral nerve ultrasonography combined with NCV calculation evoked by electrical stimulation makes it possible to further evaluate *in vivo* human peripheral nerve morphology and function.

Unlike the upper limbs, there have only been a few reports of NCV for the lower limbs, especially for a comparison of NCV for both the upper and lower limbs (Kim et al., 2009b). From the literature, the NCV of the upper limbs would be higher than that of the lower limbs (Mayer, 1963). However, the peripheral nerve ultrasonography showed that the lower limbs had greater nCSA than the upper limbs (Bedewi et al., 2017, 2018). These NCV and nCSA reports are not in line with the principle that the greater the peripheral nerve axon diameter, the higher the NCV (Edds, 1950; Samorajski and Rolsten, 1975). This conflict needs to be thoroughly examined during the simultaneous comparison between both human upper and lower limbs and at different regions because nCSA was not uniformly developed from the distal to proximal parts (Nobue and Ishikawa, 2015). The regional specificity of the nCSA developments and functions in both the upper and lower limbs also need to be fully discussed. In addition, the postural lower limb muscles had greater innervation ratio calculated by the number of muscle fibers dominated by axons than in the upper limb fine regulator muscles (Feinstein et al., 1955). Therefore, the branch unit of efferent nerve fibers in the greater innervation ratio muscle could be greater nerve axon size, and therefore, the lower limbs could have greater nCSA than the upper limbs. In this case, the simultaneous NCV measurements for both the upper and lower limbs together with nCSA measurements can solve the above-mentioned discrepancies and demonstrate the existence of the limb-specific NCV profiles.

The specificities of the lateral preference and dominancy of the upper limbs have been well examined but not in the lower limbs. McGrath et al. (2015) suggested the difficulty to discern the lateral preference and dominancy of the lower limbs. A previous human lower limb study (Kim et al., 2009b) found no differences in the NCV of the lower limbs between the dominant and non-dominant sides. Therefore, the lower limbs could have smaller differences in the nCSA and muscle sizes between the dominant and non-dominant sides than in the upper limbs. Therefore, unlike the upper limbs, the lateral preference and dominancy of the lower limbs may not exist.

Therefore, this study aimed to simultaneously examine the nCSA size of the upper and lower limb regions with the NCV of the ulnar and tibial nerves. Our hypotheses are as follows: (1) NCV, nCSA, and limb circumference are greater in the lower limbs than in the upper limbs. (2) The nCSA at any upper and lower limb region depends upon their circumferences. However, the lower limb shows no significant correlation between the size of the lower limb nCSA and its NCV as is the case with the previous upper limb study (Nobue and Ishikawa, 2015). Functionally, the proximal parts of the limbs have higher and thinner NCV and nCSA and *vice versa* than the distal parts. (3) Unlike the upper limbs, the NCV and nCSA of the lower limbs do not show any lateral preference despite the varying circumferences of both legs.

## Materials and Methods

### Subjects

Thirty participants who have no history of any neurological, peripheral neuropathy, or other disorders of the upper and lower limbs, as well as no bilateral differences of the forearm and shank length, volunteered for this study [25 male and 5 female; age 19.8 ± 1.6 (18–25) years; body mass 65.5 ± 15.6 kg; height 172.0 ± 6.7 cm]. All subjects were competitive and active athletes who regularly attended local competitions for more than 6 years [tennis, baseball, track and field (sprint, javelin throw, high jump, hurdle, and decathlon), rugby, or soccer]. The dominant hand was confirmed by the Edinburgh Handedness Inventory (Oldfield, 1971), and the dominant (reacting) and non-dominant (supporting) legs were confirmed by the Waterloo Footedness Questionnaire (Elias et al., 1998). Informed consent was obtained before the experiment, which was conducted according to the guidelines of the Declaration of Helsinki and was approved by the Ethics Committee of the Osaka University of Health and Sport Sciences (authorization number 19-8).

### Protocols

Firstly, the upper and lower limb circumferences were measured using a measuring tape. The nCSA of the ulnar and tibial nerves of the participants were measured by ultrasonography [Noblus, Hitachi Aloka Medical Ltd., a high-frequency (18 MHz) linear array ultrasound transducer; image resolution: 0.08 mm] in the sitting position with the forearm flexed at 120° and in the abdominal position, respectively. After the nCSA measurements, NCVs of the ulnar and tibial nerves were measured using the standard techniques of supramaximal percutaneous stimulation with a constant current stimulator (DS7A, Digitimer Ltd., United Kingdom) and surface electrode recording (P-EMG plus, Oisaka Electronic Equipment, Japan) on each limb of each subject.

### Measured Parameters

#### Nerve Cross-Sectional Area

**Figure 1:**
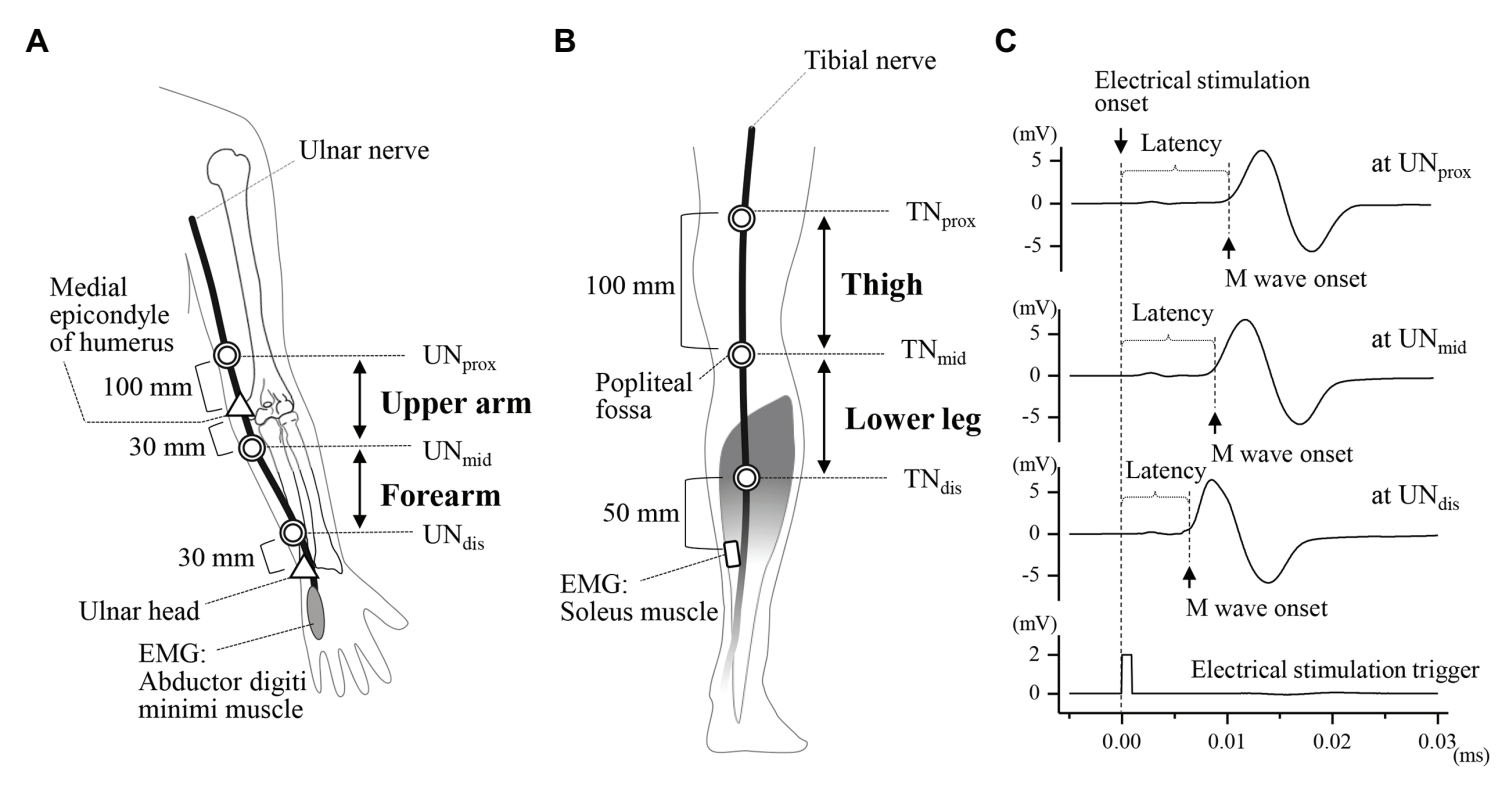
The measurement setup for the ulnar and tibial nerves and the representative EMG responses with the compound muscle action potentials of the measured points of the cross-sectional area (CSA) and positions of nerve electrical stimulation of the ulnar and tibial nerves. **(A)** The measured positions of the circumstance, nerve cross-sectional area (nCSA), and electrical stimulation for the ulnar nerve: 100 mm proximal to the medial epicondyle of the humerus (UN_prox_), 30 mm distal to the medial epicondyle of the humerus (UN_mid_), and 30 mm proximal to the ulnar head (UN_dis_). **(B)** The measured positions of the circumstance, nCSA and electrical stimulation for the tibial nerve: 100 mm proximal to the popliteal fossa (TN_prox_), at the popliteal fossa (TN_mid_), and 50 mm proximal to the soleus muscle (TN_dis_). **(C)** The onset of the M wave was measured by the compound muscle action potential recorded from each stimulation point.

The ulnar and tibial nerves were scanned at three regions in the upper and lower limbs, respectively (Figures 1A,B). In the upper limb, the first region was at 100 mm proximal point to the medial epicondyle of the humerus (UN_prox_), the second region was at 30 mm distal point to the medial epicondyle of the humerus (UN_mid_), and the third region was at 30 mm proximal point to the ulnar head (UN_dis_). In the tibial nerves, the first region was at 100 mm proximal point to the popliteal fossa (TN_prox_), the second region was at the popliteal fossa point (TN_mid_), and the third region was at 50 mm proximal point to the soleus muscle belly (TN_dis_). As mentioned above, the nCSA size, which is a bundle of nerve fibers was measured by the ultrasonographic images at each region of the ulnar and tibial nerves, respectively (Nobue and Ishikawa, 2015). From these ultrasonographic images, the boundary of the nerve circumference was traced, and the upper and lower limb nCSAs were separately analyzed at each point (UN_prox_, UN_mid_, and UN_dis_; TN_prox_, TN_mid_, and TN_dis_, respectively) by ImageJ software (ver 1.45 s, National Institutes of Health, Unites States). The mean upper and lower limb nCSAs were calculated by the measured three points at each limb, respectively. For the comparison between the different regions, the upper arm and forearm nCSAs were averaged by nCSA at UN_prox_ and UN_mid_ and at UN_mid_ and UN_dis_, respectively. Moreover, the thigh and lower leg nCSAs were averaged by nCSA at TN_prox_ and TN_mid_ and at TN_mid_ and TN_dis_, respectively.

#### Upper and Lower Limb Circumferences

The circumferences of the upper and lower limbs were measured around the maximal girth of the forearm and calf and at the measured nCSA points of UN_prox_, UN_mid_, UN_dis_, TN_prox_, TN_mid_, and TN_dis_, respectively (Figures 1A,B). The CSA for each arm and leg region was calculated from each circumference:

$$\mathrm{CSA}\;\mathrm{at}\;\mathrm{each measured point}=\frac{1}{4\pi }\;{\left(each\;circumference\right)}^{2}$$

The upper arm and forearm circumferences were averaged by circumferences at UN_prox_ and UN_mid_, and at UN_mid_ and UN_dis_, respectively. Furthermore, the thigh and lower leg circumferences were averaged by circumferences at TN_prox_ and TN_mid_, and at TN_mid_ and TN_dis_, respectively.

#### Motor Nerve Conduction Velocity

The motor response from each muscle was collected using the Signal software (Signal version 7.01, Cambridge Electronic Design Limited, United Kingdom) at a sampling rate of 10 kHz (Power 1,401, Cambridge Electronics Design Limited, United Kingdom). The compound muscle action potentials (CMAPs) were evoked by the electrical stimulation (DS7A, Digitimer Ltd., United Kingdom; 0.2 ms duration constant current square wave pulses) of the ulnar and tibial nerves starting from minimal and progressing to supramaximal stimuli intensity. As shown in Figure 1A, the CMAPs are evoked from the abductor digiti minimi muscle after electrical stimulation of ulnar nerve at the same nCSA measured points. The active electrode was attached to the belly of the abductor digiti minimi muscle (the muscle innervated by the ulnar nerve) and a ground electrode was attached to the ulnar head. Examination was performed with the subjects sitting and the forearm flexed at 120°. Similarly, as shown in Figure 1B, the CMAPs are evoked from the soleus muscle after electrical stimulation of tibial nerve at the same nCSA measured points. The active electrode was attached to the belly of the soleus muscle (the muscle innervated by the tibial nerve) and a ground electrode was attached to the malleolus lateralis. Examination was performed with the subjects lying prone and the ankle in a neutral position. The stimulation regions for both upper and lower limbs were marked with an aqueous marker, and the distances between stimulation regions were measured using a measuring tape. The latency of the stimulus artifact at each point was detected as the onset of the M-wave (Figure 1C). NCV was calculated by dividing the distance between each stimulating point by the differences between the latency responses (Kimura, 2013). These values were used as follows:

**Upper arm NCV of the ulnar nerve**: the distance from UN_prox_ to UN_mid_, the latency from UN_prox_ to UN_mid_.

**Forearm NCV of the ulnar nerve**: the distance from UN_mid_ to UN_dis_, the latency from UN_mid_ to UN_dis_.

**Upper limb NCV of the ulnar nerve**: the average value of these above two NCVs.

**Thigh NCV of the tibial nerve**: the distance from TN_prox_ to TN_mid_, the latency from TN_prox_ to TN_mid_.

**Lower leg NCV of the tibial nerve**: the distance from TN_mid_ to TN_dis_, the latency from TN_mid_ to TN_dis_.

**Lower limb NCV of the tibial nerve**: the average value of these above two NCVs.

Skin and core body temperatures (around the soleus and abductor digiti minimi muscles) of each subject were monitored (CORE, greenTEG AG, Switzerland) at each trial to avoid the influence of temperature on NCV. During measurements, we confirmed that the skin and core body temperatures stayed constant at each subject.

### Statistical Analyses

Results were presented as means ± standard deviations. For comparison between the upper and lower limbs, the mean maximal circumferences, NCVs and nCSA for each subject were averaged for the right and left arms and for the right and left legs, respectively. Prior to all statistical analyses for this comparison, the distribution of the variables was passed for normality. Thus, statistical analyses were performed using the paired *t*-test between the upper and lower limbs. For the lateral comparison, the variables were compared between dominant and non-dominant arms as well as between the supporting and reacting legs, respectively. For comparison between regions for each upper and lower limb, the variables were compared between forearm and upper arm and between thigh and lower leg, respectively. To consider the statistical test of interaction between these two comparisons, Mauchly’s test of sphericity was performed on the data, and a two-way repeated-measures ANOVA (rmANOVA) was used to test for significance of the main effects of each parameter and interaction between lateral comparison as well as regions for the upper and lower limbs, respectively. When no transform was found that made the variable normally distributed, nonparametric Wilcoxon signed rank tests were used to test for differences between groups and the significance levels were Bonferroni corrected. The correlations between each parameter were evaluated using the Pearson’s correlation coefficients after the distribution of the variables was passed for normality. The confidence level was set at *p* < 0.05 to determine statistical significances for all data. SPSS 25.0 software was used for statistical analyses.

## Results

For comparison between upper and lower limbs (averaged variables between lateral parts), Table 1 shows the maximum circumference, NCV, and nCSA, respectively. The lower limb had significantly greater maximum circumferences, NCV, and nCSA than the upper limb, respectively (*p* < 0.05).

**Table 1:** Comparison of the measured parameters for the upper and lower limbs.

	Upper limb Ulnar nerve	Lower limb Tibial nerve
Maximum circumferences (mm)	260 ± 30	374 ± 18^*^
Motor nerve conduction velocity (m s^−1^)	55.6 ± 4.3	59.1 ± 9.0^*^
Nerve cross-sectional area (mm^2^)	6.6 ± 1.2	23.3 ± 4.1^*^

Values are expressed as means ± SD. The ulnar and tibial nCSAs were averaged by the three measured points for both the dominant and nondominant arms and for both the supporting and reacting legs, respectively.

* Shows significant differences between the upper and lower limbs (*p* < 0.05).

For the lateral comparison at upper limbs (averaged variables between upper arm and forearm), the maximum forearm circumferences, upper limb NCV, and upper limb nCSA were significantly greater in the dominant than in the non-dominant arms (264 ± 29 vs. 256 ± 31 mm, 56.7 ± 6.2 vs. 54.5 ± 4.0 m s^−1^, and 6.9 ± 1.6 vs. 6.2 ± 1.2 mm, respectively: *p* < 0.05). For the lateral comparison at lower limbs (averaged variables between thigh and lower leg), the maximum lower leg circumference was greater in the supporting than in the reacting legs (376 ± 19 vs. 373 ± 18 mm, *p* < 0.05). The tibial nerve nCSA did not show any significant differences between the supporting and reacting legs (23.1 ± 4.7 vs. 23.6 ± 5.0 mm^2^, respectively). However, the mean lower limb NCV was significantly lower in the supporting than in the reacting legs (55.7 ± 11.3 vs. 62.5 ± 10.7 m s^−1^, *p* < 0.05).

**Figure 2:**
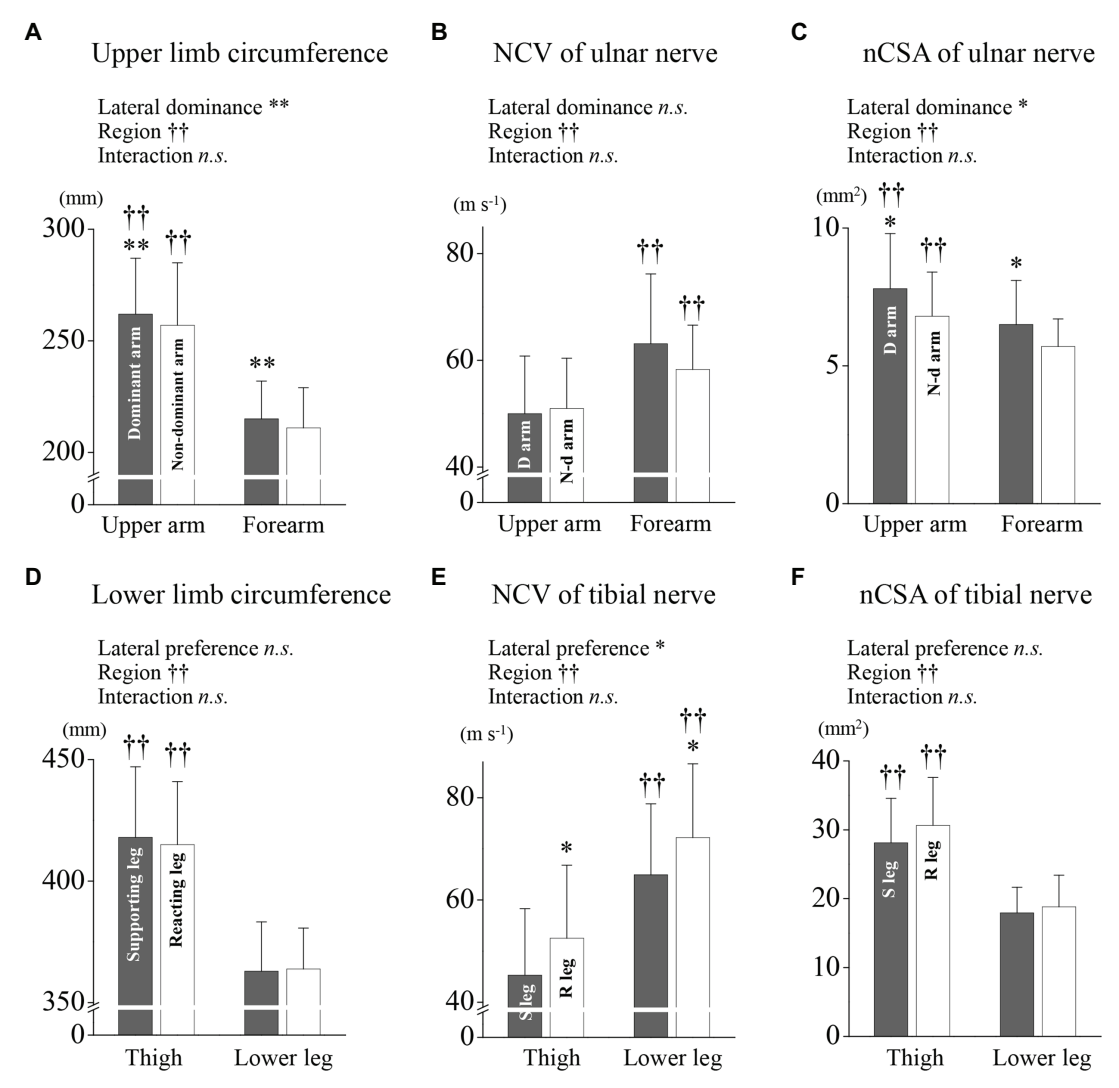
Limb circumference, nerve conduction velocity (NCV), and nCSA nerve cross-sectional area for the upper and lower limbs. **(A)** The upper arm and forearm arm circumferences for the dominant and non-dominant arms are shown, respectively. **(B)** The ulnar NCV of the upper arm and forearm are shown for the dominant and non-dominant arms, respectively. **(C)** The ulnar nCSA of upper arm and forearm are shown for the dominant and non-dominant arms, respectively. **(D)** The thigh and lower leg circumferences for the supporting and reacting legs are shown, respectively. **(E)** The tibial NCV of the thigh and lower leg are shown for the supporting and reacting legs, respectively. **(F)** The tibial nCSA of the thigh and lower leg are shown for the supporting and reacting legs, respectively. ^*^, ^†^, and ns indicate repeated two-way ANOVA analysis showing the main effect of lateral dominance (preference), region and interaction. ^*^, ^**^, and ^††^ are significantly higher values as compared with the others (^*^p < 0.01, ^**^p < 0.001, ^††^*p* < 0.001, ns, not significant), respectively.

More detail comparisons were performed for examining region and lateral specificities. In the circumference of the upper limb, the rmANOVA with lateral dominance and region as factors showed no interaction between all variables and revealed main effects of lateral dominance [*F*_(1,29)_ = 19.54, *p* < 0.001] and region [*F*_(1,29)_ = 521.42, *p* < 0.001], respectively (Figure 2A). In the ulnar NCV, the rmANOVA with lateral dominance and region as factors showed no interaction between all variables and revealed main effects of region [*F*_(1,29)_ = 18.90, *p* < 0.001] but not lateral dominance [*F*_(1,29)_ = 2.891, *p* = 0.100], respectively (Figure 2B). In the ulnar nCSA, the rmANOVA with lateral dominance and region as factors showed no interaction between all variables and revealed effects of lateral dominance [*F*_(1,29)_ = 10.42, *p* = 0.003] and region [*F*_(1,29)_ = 29.19, *p* < 0.001], respectively (Figure 2C).

In the circumference of the lower limb, the rmANOVA with lateral preference and region as factors showed no interaction between all variables and revealed main effects of region [*F*_(1,29)_ = 295.83, *p* < 0.001] but not lateral preference [*F*_(1,29)_ = 0.34, *p* = 0.564], respectively (Figure 2D). In the tibial NCV, the rmANOVA with lateral preference and region as factors showed no interaction between all variables and revealed effects of lateral preference [*F*_(1,29)_ = 10.26, *p* = 0.003] and region [*F*_(1,29)_ = 56.71, *p* < 0.001], respectively (Figure 2E). In the tibial nCSA, the rmANOVA showed no interaction between all variables and revealed with lateral preference and region as factors showed no interaction between all variables and revealed main effects of region [*F*_(1,29)_ = 213.99, *p* < 0.001] but not lateral preference [*F*_(1,29)_ = 3.526, *p* = 0.078], respectively (Figure 2F).

**Figure 3:**
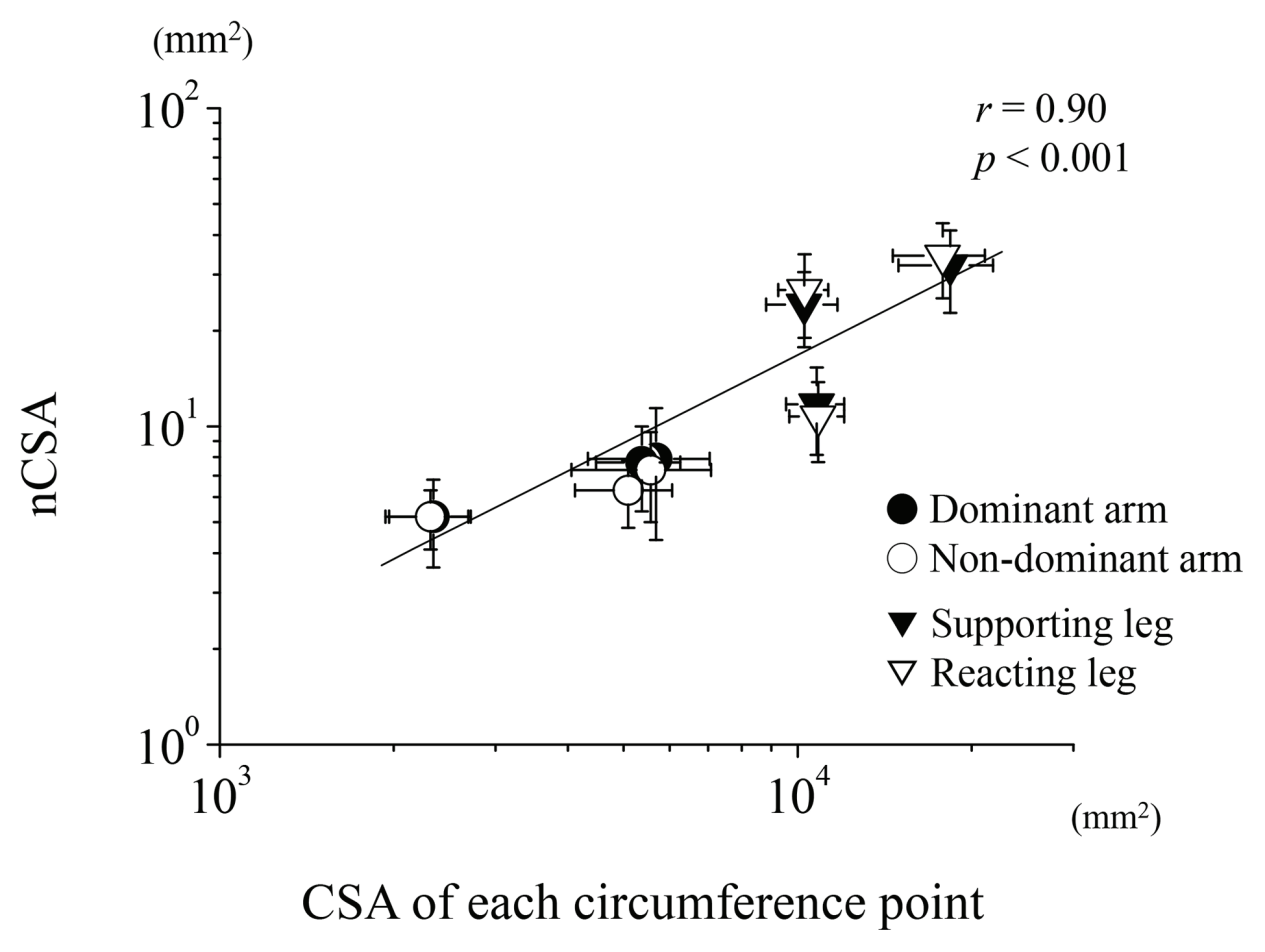
Relationship between the nerve and CSA for the upper and lower limbs. Semi-log plots of nCSA and the estimated CSA of each circumference are plotted for both the upper and lower limbs.

Figure 3 shows a semi-log plot of the nCSA at each region vs. the CSA calculated from its circumference at each region of the upper and lower limbs. According to the Pearson’s correlation, the data of all limbs clearly follow a straight line, which indicates that all nCSA data of both limbs maintain a relatively constant value to its circumference (Figure 3; *r* = 0.90, *p* < 0.001), although the tibial nerve had a much greater nCSA than the ulnar nerve.

**Figure 4:**
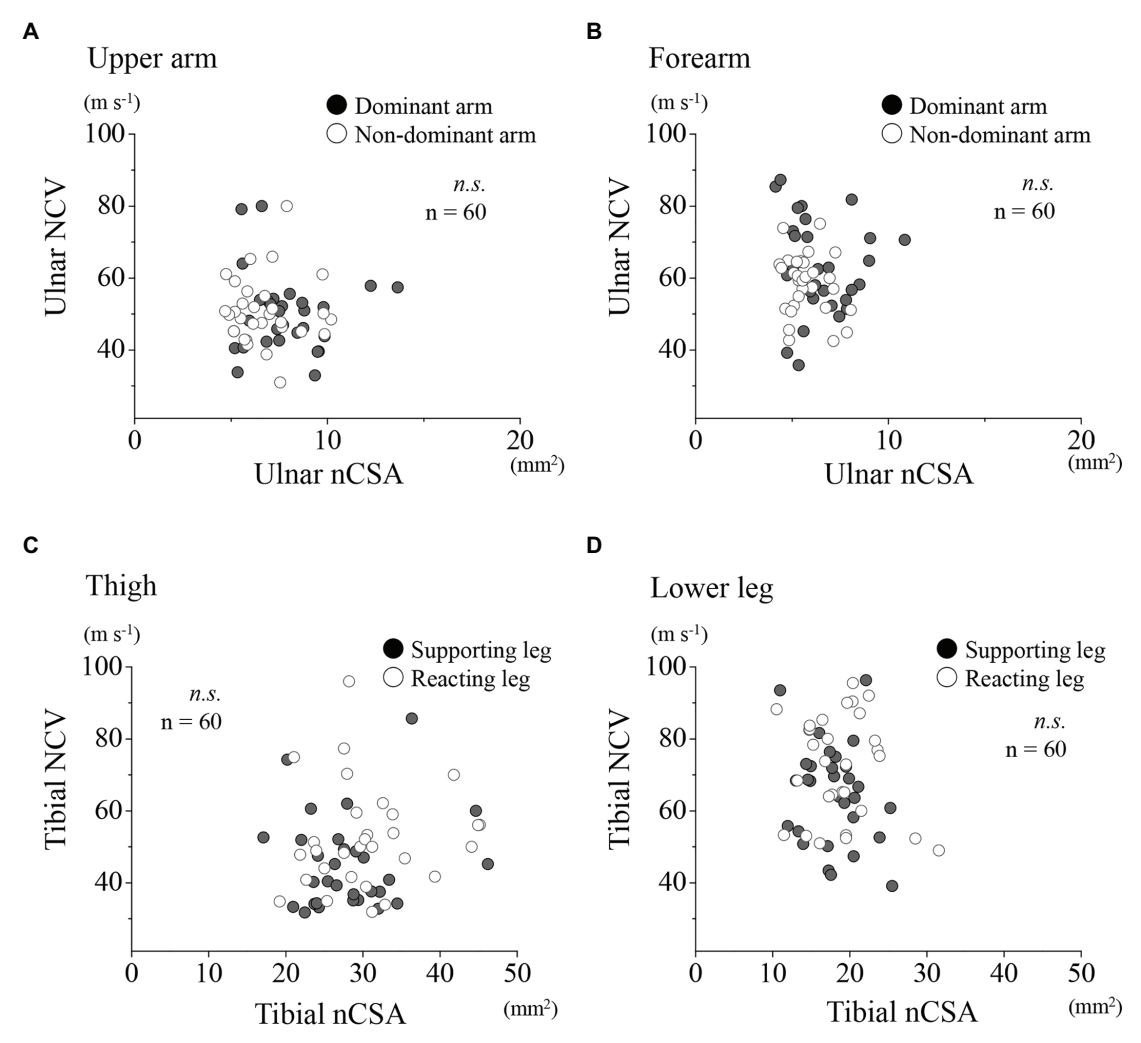
Relationships between motor NCV and nCSA for the upper and lower limbs, respectively. Relationships between motor NCV and nCSA of the upper arm **(A)** and forearm **(B)** for the dominant (●) and non-dominant arms (○) as well as of the thigh **(C)** and lower leg **(D)** for the supporting (●) and reacting legs (○).

**Figure 5:**
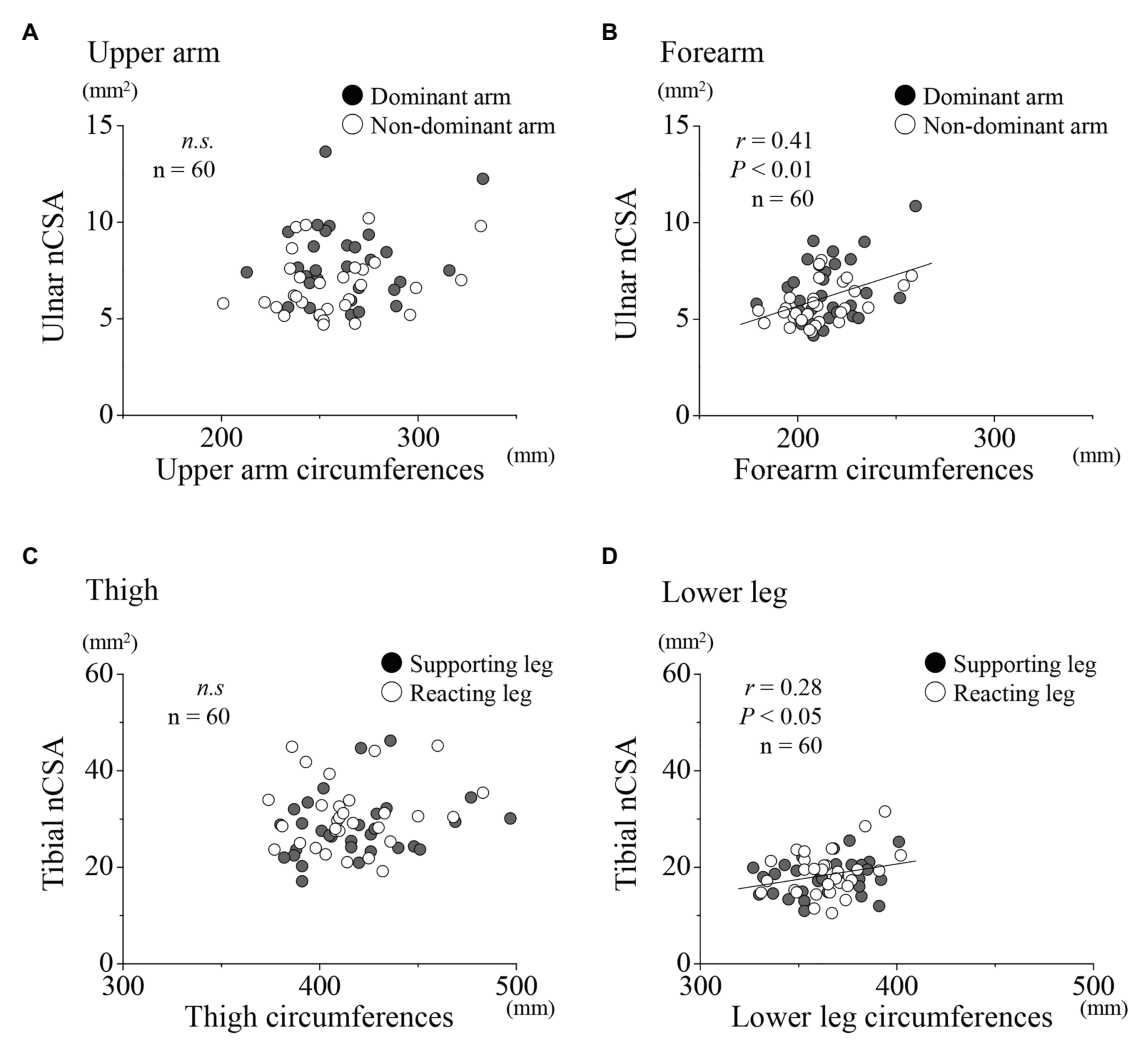
Relationships between nCSA and circumferences for the upper and lower limbs, respectively. Relationships between nCSA of the upper arm **(A)** and forearm **(B)** for the dominant (●) and non-dominant arms (○) as well as of the thigh **(C)** and lower leg **(D)** for the supporting (●) and reacting legs (○).

A further examination of the relationships between the ulnar NCV and nCSA at each region for the upper limbs showed no significant correlation for both dominant and non-dominant arms together, and for the dominant and non-dominant arms, respectively (Figures 4A,B). Similarly, at the lower limbs, no significant correlation was found between the tibial nerve NCV and nCSA (Figures 4C,D). In the upper limbs, positive correlations were found between the forearm nCSAs and the forearm circumferences (Figure 5B; *r* = 0.41, *p* < 0.01). In the lower limbs, a weak positive correlation was found between the lower leg nCSAs and their circumferences (*r* = 0.28, *p* < 0.05; Figure 5D).

## Discussion

Our results clearly showed that the lower limbs had higher and greater NCV, nCSA, and circumference than the upper limbs. However, NCV did not show any relationships with nCSA and circumference for both the upper and lower limbs. Unlike the upper limbs, the reacting leg had higher NCV than the supporting leg, despite the supporting leg having greater circumference than the reacting leg. Therefore, the absolute nCSA size can be related to its circumference but not necessarily function to the NCV developments for the limbs. These varying aspects between the upper and lower limbs indicate the existence of limb-specific NCV developments but not nCSA developments.

### Limb Specificity of Neuromuscular Features

As surmised by the reports of Bedewi et al. (2017, 2018), our results clearly confirmed that the lower limbs had an approximately 3.5 times greater human nCSA than the upper limbs, despite the lower limbs having approximately 1.4 times greater maximum circumference than the upper limbs. Additionally, the lower limbs NCV (59.1 ± 9.0 m s^−1^) was greater than that in the upper limbs (55.6 ± 4.3 m s^−1^). This result does not necessarily coincide with those of the previous study, which showed that the lower limbs had lower NCV (45.5 ± 3.8 m s^−1^) than the upper limbs (58.9 ± 2.2 m s^−1^; Mayer, 1963). This conflicting result could be related to the testing place of the leg muscles and nerves, which were much more distal in the previous study (abductor hallucis muscle and tibial nerve) than in the present study (soleus muscle and tibial nerve). Additionally, the NCV measured in the proximal part of the upper limb can be higher than that in the distal part (Trojaborg and Sindrup, 1969). Another possibility of the conflicting result is the influence of the tested subjects. The present study had active athletes as participants; however, those in the previous study were not (mentioned as just normal subjects). The NCV of the active athletes may be developed compared with the normal healthy subjects. When taken together, our results clearly showed that the lower limbs had greater and higher nCSA and NCV than the upper limbs. However, all nCSA data of each region in both the upper and lower limbs maintain a relatively constant value to its circumference and NCV did not show any relationships with nCSA for both the upper and lower limbs. Therefore, these aspects of NCV and nCSA between the upper and lower limbs indicate the existence of limb-specific NCV characteristics and non-limb-specific nCSA developments.

### Lateral Preferences of Neuromuscular Features in the Upper and Lower Limbs

As shown by the previous study (Nobue and Ishikawa, 2015), the present study confirmed that the dominant arms had greater circumference and nCSA than the non-dominant arms. Furthermore, the dominant arm had higher NCV than the non-dominant arms. Therefore, the dominant upper limb can have greater and higher nCSA and NCV than the non-dominant upper limb, respectively. In the lower limbs, however, the supporting leg had lower NCV than the reacting leg. In addition, the nCSA cannot be necessarily high in the big supporting leg compared with the small reacting leg. In the reaction movements of the lower limbs, not only the reacting but also the opposite supporting legs work as the inherent functions of supporting the body weight prior to performing movements, which requires effective coordination between both legs (Promsri et al., 2020). Meanwhile, the reaction movements of the upper limbs could be focused by testing one arm but not another arm. Thereby, unlike the upper limbs, both lower limbs can have different functions to the reaction movements. Thus, the limb-specific function to quick response movements may lead to different results of lateral preferences of neuromuscular features for both the upper and lower limbs, respectively.

### Region Specific of Peripheral Nerve Features

Trojaborg and Sindrup (1969) reported that NCV measured in the proximal part of upper limb was higher than that in the distal part. In this case, the nerve axon diameter may be possible to be greater in the proximal part than in the distal part due to the less nerve branching in the proximal part. However, there have not been any reports about the comparison of the nerve axon diameter at different regions. In the nCSA level of the present study, the nCSA in the proximal parts were greater than that in the distal parts for both the upper and lower limbs, depending on their circumferences (Figures 2, 5). However, the distal parts of both limbs had higher NCV than the proximal parts. These results were not in line with those of the upper limbs in the previous *in vivo* study (Trojaborg and Sindrup, 1969). Further examination of the region specific NCV and nerve size in both the upper and lower limbs are needed to solve this inconsistency. Taken together, the present study suggests that the nCSA at any regions in both the upper and lower limbs can be depended on their circumferences. In addition, our results imply the region-specificity in both the upper and lower limbs, where the distal parts of limbs can have higher NCV as well as thinner nCSA and *vice versa* than the proximal parts.

### Methodological Limitations

The diameter size of the peripheral nerve fiber is proximately 10–30 μmm. So far, the resolution of current *in vivo* human imaging technology cannot identify an axon diameter of the nerve fibers. Therefore, in the present study, we have measured the size of the nerve trunk (nCSA), which is a bundle of nerve fibers, using peripheral nerve ultrasonography. In this size level, nCSA could contain a variety of fiber types, not only the efferent fibers but also afferent fibers. Therefore, further considerations in the diameter level of the myelinated nerve fibers and the distinction of the efferent fibers from different fiber types of mixed peripheral nerve should be given in future studies with more detail high-resolution ultrasonography. In this study, the subject was selected with no bilateral differences of the forearm and shank length and was recruited to minimize the effects of the different distances between the nodes of Ranvier in the myelinated axons. Further validation needs to expand the various subject groups.

## Conclusion

Direct measurements of the human NCV and nCSA clearly showed the morphological and functional differences between the upper and lower limbs and between regions of both limbs. The different aspects between the upper and lower limbs and between regions suggest that NCV does not depend on either the nCSA sizes or circumference of both upper and lower limbs and indicate the existence of limb-specific NCV developments but not nCSA developments.

## Data Availability Statement

The original contributions presented in the study are included in the article/supplementary material, further inquiries can be directed to the corresponding author.

## Ethics Statement

The studies involving human participants were reviewed and approved by the Human Ethics Committee of Osaka University of Health and Sport Sciences. The patients/participants provided their written informed consent to participate in this study.

## Author Contributions

AN, YK, KS, HO, and MI contributed to the study concept and design. AN, KS, HO, and MI contributed to methodological developments. AN, YK, and HT carried out the experiment, the data analysis, and interpretation. AN and MI co-wrote the paper. All authors contributed to the article and approved the submitted version.

### Conflict of Interest

The authors declare that the research was conducted in the absence of any commercial or financial relationships that could be construed as a potential conflict of interest.
